# Historical domestication-driven population expansion of the dung beetle *Gymnopleurus mopsus* (Coleoptera: Scarabaeidae) from its last refuge in Mongolia

**DOI:** 10.1038/s41598-018-22182-3

**Published:** 2018-03-02

**Authors:** Ji Hyoun Kang, Chang Seob Lim, Sung Hwan Park, Sang Woo Seok, Tae Joong Yoon, Badamdorj Bayartogtokh, Yeon Jae Bae

**Affiliations:** 1 Korean Entomological Institute, College of Life Sciences and Biotechnology, Korea University, Seoul, Korea; 2 Department of Environmental Science and Ecological Engineering, Graduate School, Korea University, Seoul, Korea; 3 School of Arts and Sciences, National University of Mongolia, Ulaanbaatar, Mongolia

## Abstract

Populations of *Gymnopleurus mopsus* (family Scarabaeidae), a dung beetle that displays dung-rolling behavior (i.e., a telecoprid), have recently experienced sharp declines, and many populations are now at high risk of local extinction. However, Mongolia, which constitutes a major portion of the species’ distribution, still sustains a relatively large population. Here, we used mitochondrial *COI* sequences to investigate the within-population genetic diversity and both the genetic and phylogeographic structures of 24 *G*. *mopsus* populations across the species’ main distribution in Mongolia. Several lines of evidence indicated that the phylogeographic structure of *G*. *mopsus* had been influenced by a recent and sudden demographic expansion. Interestingly, the expansion of Mongolia’s *G*. *mopsus* population corresponded to the advent of livestock domestication in the region, and the species’ genetic structure coincided with road networks, which presumably serve as migration routes for livestock that might mediate the beetle’s dispersal. In addition, we also found that *G*. *mopsus* possesses high levels of haplotype diversity, which is generally indicative of large effective population sizes (*N*_e_). Overall, the present study contributes to the current understanding of *G*. *mopsus*’ demographic history and dispersal patterns and also provides valuable information for the species’ conservation and management.

## Introduction

Copronecrophagous dung beetles (subfamily Scarabaeinae) possess specialized ecological ability and behavior, in that they utilize feces as food, habitat, and breeding ground, and this extraordinary case of ecological specialization has likely contributed to their evolutionary success^1^. These dung beetles are also thought to play important ecological roles, such as nutrient recycling, bioturbation, plant growth promotion, seed dispersal, and parasite control, in terrestrial ecosystems^2,3^ and, as a result, have become a focus of both biodiversity and conservation research, especially owing to their broad range of responses to environmental changes and strong ecological interactions with a diverse array of organisms, including plants, vertebrates, and invertebrates^4^.

Unfortunately, however, both the decreased of population size and local extinction of dung beetle species (extinction-prone) have been reported to occur as a result of natural and anthropogenic disturbances^2,3,5,6^. It has been suggested that the diversity and abundance of dung beetles are strongly affected by slight changes in land use and mammalian fauna, at even local scales. Overhunting of forest-dwelling mammals, for example, has led to the extirpation of dung beetle species that rely on the dung of particular mammals^2,7^. In addition, dung beetle species with rolling behavior (i.e., telecoprids) are especially vulnerable to anthropogenic activities, and historical records suggest that the relative frequencies of telecoprid species have been more severely affected than other scarab dung beetles during the 20^th^ century^8^. Furthermore, the use of antibiotics and parasite treatments (e.g., ivermectin) in commercial livestock management and agriculture can also accelerate the decline and extinction of dung beetle species^9,10^, thereby profoundly affecting community- and ecosystem-level changes.

Similar to other telecoprid species, *Gymnopleurus mopsus* (Scarabaeidae), is also under threat of local extinction throughout its distribution in Palearctic regions, including in Asia^11^ and Europe^6,8^. Previous studies have reported that both *G*. *mopsus* and other roller species are threatened in Italy^8^ and that populations of *G*. *mopsus* on the Iberian Peninsula have declined significantly^6^. In addition, *G*. *mopsus* has also been declared as regionally extinct (last documented in the 1960s) on the Korean Peninsula, which constitutes the southern margin of the species’ range in Northeast Asia. As a result, the National Institute of Biological Resources in South Korea launched a restoration program in 2012, which re-introduced *G*. *mopsus* from Mongolia to South Korea, based on the high genetic diversity of the Mongolia population and its genetic similarity to populations in South Korea^12^.

Because Mongolia is the only region where large *G*. *mopsus* populations are currently maintained in the north-eastern Palearctic region^11^, it has the value as the species’ last “refuge” and, as such, is considered important for future conservation and restoration efforts. However, the present-day population genetic structure shaped by contemporary processes (e.g., ongoing gene flow among populations in relation to anthropogenic habitat fragmentation and distributional changes) as well as demographic (population) history (e.g., dispersal, isolation, population contraction/expansion and bottleneck) of *G*. *mopsus* in Mongolia are yet to be investigated.

Such investigations of Mongolian *G*. *mopsus* populations would allow an understanding of the various ecological and evolutionary aspects of *G*. *mopsus*. The dispersal and migration patterns of insect species have been reported to play essential roles in determining the genetic structure of insect populations^13^, and the ecological associations of dung beetles and mammals have likely contributed to the evolution of the Scarabaeinae and community structuring of extant dung beetles over the last 40 million years^14^. Therefore, analysis of both population genetic and phylogeographic structures could elucidate the historic migration, dispersal, and demography, and the *G*. *mopsus* populations in Mongolia present a promising model system for understanding the effects of mammal (e.g., cattle, sheep, and camels) abundance and distribution on the animal-mediated dispersal patterns of dung beetles.

The current population genetic structure of a species is shaped by the effects of the population demographic history (i.e., evolutionary history) of a species, as well as the level of contemporary gene flow among populations. Therefore, historical anthropogenic events, such as the domestication of wild animals, could have influenced the population demography of dung beetles. Studying the levels of genetic diversity and population genetics structure of *G*. *mopsus* in Mongolia would allow us to infer whether anthropogenic activities, such as moving cattle herds by pasturing, have facilitated the dispersal of dung beetles. Phylogeographic approaches can also reveal the historical and evolutionary origins and demographic processes of *G*. *mopsus*, including the genetic footprints of historic fluctuations in population demography, which could also be related to human activities^15^. Phylogeographic analysis might also elucidate the effects of human-mediated disturbances, such pasture reduction or habitat destruction, on the distribution and size of *G*. *mopsus* populations. Pasturing areas in Mongolia have been rapidly decreased by urbanization and changes to pasturing style. Livestock pasturing can prevent the range expansion of some dung beetle species ranges by altering vegetation density, temperature, and moisture levels^16^. Mongolia can be classified into two large biogeographic regions: northern humid areas (grassland and forest-steppe areas) and southern desert or semi-desert areas that have undergone drastic climate changes seasonally^17^. Therefore, determining both the current population genetic and phylogeographic structures of *G*. *mopsus* may help us to elucidate its historical distribution patterns in an ecological context.

Although *G*. *mopsus* currently maintains large populations in Mongolia, future population declines are very likely, owing to rapid urbanization and land development (e.g., mining, road construction etc.) now taking place in Mongolia. Here, we first investigated the population genetic and phylogeographic structures of *G*. *mopsus* in Mongolia, in order to understand the species’ evolutionary history (e.g., the pattern of migration, population contraction/expansion) and distribution, as well as to determine its current population genetic status.

## Results

### Mitochondrial diversity

**Figure 1:**
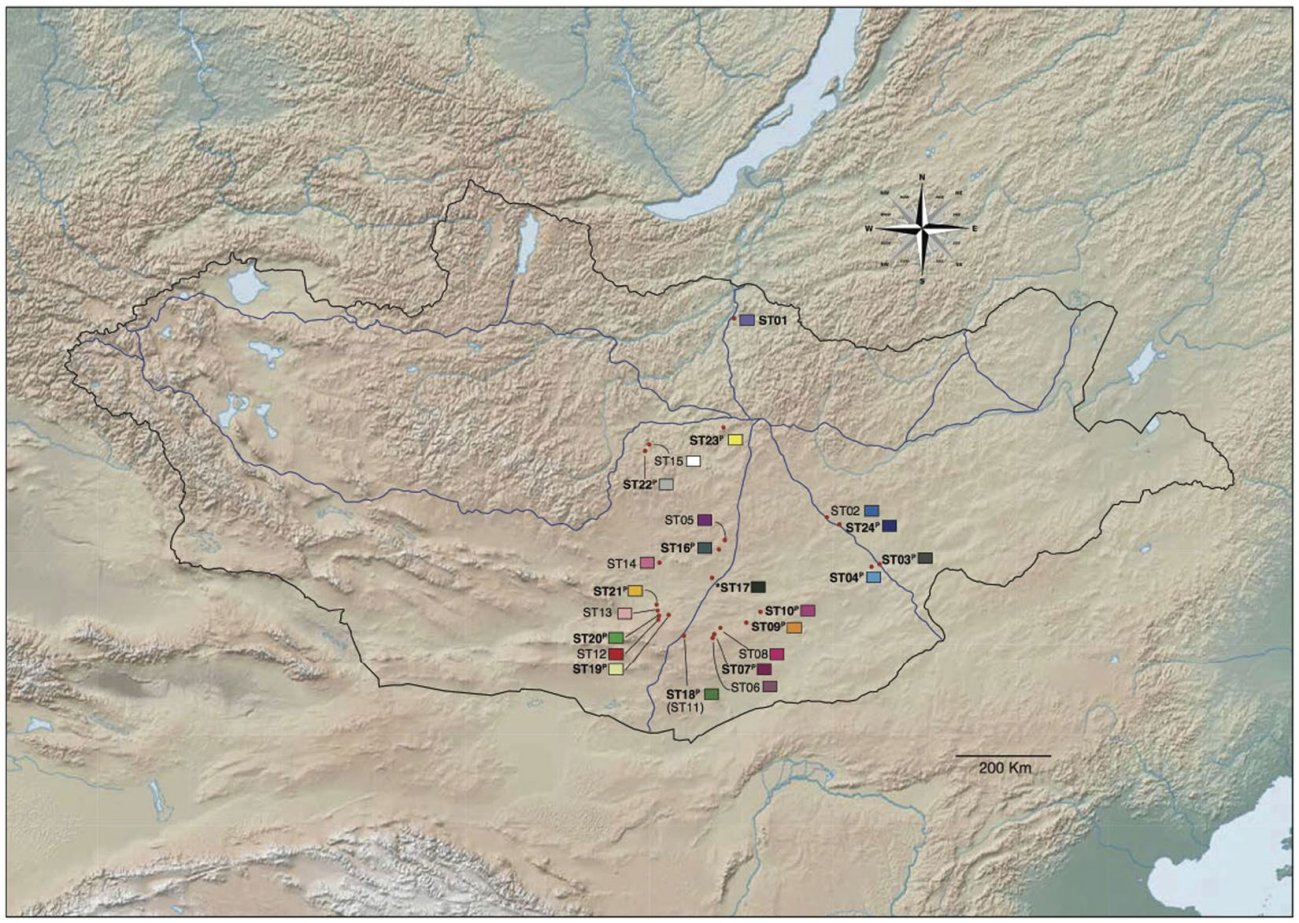
Localities of *Gymnopleurus mopsus* samples collected in Mongolia. The map of Mongolia was modified from a map produced using ArcMap 10.5^52^ (www.esri.com) with the World Terrain Basemap (Esri, USGS, NOAA). Each color represents 24 different sampling localities. The 14 populations (N > 10) used for the present study’s population genetics analyses are indicated in bold.

Among 418 *COI* sequences (658 bp) that were generated from *G*. *mopsus* specimens collected in Mongolia (N = 406), as well 12 specimens from Korea, China, and France, we identified 94 polymorphic sites and a total of 230 haplotypes (Table 1, Fig. 1). Only the 14 populations that were represented by more than ten samples (N > 10) were included in downstream population genetic analyses, whereas the samples from all 27 sampling localities were used for haplotype network analysis, and those from the 24 sampling localities in Mongolia were used for AMOVA (Table 1). Sequence correspond to GenBank accession numbers MF674025–MF674381.

**Table 1:** Information, localities, and diversity indices of *Gymnopleurus mopsus* samples analyzed in the present study.

Locality	Area	Year of collection	Coordinates	Region	N	No. haplotypes (N_H_)	Haplotype richness (HR)	Haplotype diversity (*h*) (SD)	Mean no. pairwise differences (SD)	Nucleotide diversity (π) (SD)	Tajima’s *D*	Fu’s *Fs*
ST01^P^	N	2014	N49°45′25.43″/E106°09'58.01″	Selenge Province	34	20	7.558	0.934 (0.030)	5.276 (2.613)	0.008 (0.004)	**−1.304**	**−7.666**
ST02	E	2014	N45°55′34.94″/E109°28′24.78″	Dornogobi Province	5	5	—	1.000 (0.127)	7.000 (3.963)	0.011 (0.007)	−0.203	−0.832
ST03	E	2014	N45°09′19.52″/E109°58′0.28″	Dornogobi Province	2	2	—	1.000 (0.500)	2.000 (1.732)	0.003 (0.004)	0.000	0.693
ST04^P^	E	2014	N45°09′11.39″/E109°58′0.03″	Dornogobi Province	34	28	9.271	0.986 (0.012)	5.332 (2.638)	0.008 (0.004)	**−1.474**(^*)^	**−22.725**
ST05	S3	2015	N45°37′29.09″/E105°55′25.53″	Dundgobi Province	1	1	—	1.000 (0.000)	0.000 (0.000)	0.000 (0.000)	0.000	0.703
ST06	S1	2015	N43°44′49.45″/E105°35′08.99″	Umnugobi Province	2	2	—	1.000(0.500)	9.000 (6.708)	0.014(0.014)	0.000	2.197
ST07^P^	S1	2015	N43°47′45.98″/E105°37′29.07″	Umnugobi Province	16	12	7.789	0.942 (0.048)	4.175 (2.190)	0.006 (0.004)	−1.374	**−5.093**
ST08	S1	2015	N43°55′55.46″/E105°47′43.64″	Umnugobi Province	1	1	—	1.000 (0.000)	0.000 (0.000)	0.000 (0.000)	0.000	0.000
ST09^P^	S1	2015	N44°02′34.10″/E106°29′54.59″	Umnugobi Province	18	16	9.281	0.987 (0.023)	4.647 (2.390)	0.007 (0.004)	−1.322	**−10.781**
ST10^P^	S1	2015	N44°14′16.51″/E106°53′59.43″	Umnugobi Province	12	11	9.167	0.985 (0.040)	5.379 (2.789)	0.008 (0.005)	−1.302	**−5.193**
ST11*	S1	2015	N43°46′50.54″/E104°47′58.17″	Umnugobi Province	1	1	—	1.000 (0.000)	0.000 (0.000)	0.000 (0.000)	0.000	0.000
ST12	S2	2015	N44°06′28.42″/E104°05′01.16″	Umnugobi Province	4	4	—	1.000 (0.177)	6.333 (3.801)	0.010 (0.007)	0.558	−0.219
ST13	S2	2015	N45°17′01.47″/E104°05′52.11″	Umnugobi Province	5	4	—	0.900 (0.161)	3.000 (1.874)	0.005 (0.003)	−0.747	−0.332
ST14	S3	2015	N45°12′53.78″/E104°07′50.87″	Dundgobi Province	7	6	—	0.952 (0.096)	4.667 (2.600)	0.007(0.005)	−1.009	−1.269
ST15	C	2015	N47°21′05.83″/E103°43′18.32″	Bulgan Province	4	2	—	0.667 (0.204)	3.333 (2.149)	0.005(0.004)	2.125	3.153
ST16^P^	S3	2016	N45°28′13.19″/E105°44′34.65″	Dundgobi Province	30	25	8.959	0.975 (0.021)	4.966 (2.485)	0.008 (0.004)	**−1.6**	**−20.085**
ST17^P^	S3	2016	N44°54′47.99″/E105°32′37.74″	Dundgobi Province	28	28	10	1.000 (0.010)	6.167 (3.022)	0.009 (0.005)	**−1.492**	**−25.264**
ST18^P*^	S1	2016	N43°46′28.95″/E104°47′29.96″	Umnugobi Province	32	29	9.59	0.992 (0.011)	5.250 (2.606)	0.008 (0.004)	−1.293	**−25.408**
ST19^P^	S2	2016	N44°10′41.30″/E104°22′13.87″	Umnugobi Province	30	29	9.874	0.998 (0.009)	6.366 (3.103)	0.010 (0.005)	**−1.476**	**−25.175**
ST20^P^	S2	2016	N44°09′38.65″/E104°05′29.37″	Umnugobi Province	30	27	9.621	0.993 (0.011)	4.885 (2.449)	0.007 (0.004)	**−1.802**	**−25.075**
ST21^P^	S2	2016	N44°22′33.54″/E104°02′32.60″	Umnugobi Province	28	27	9.854	0.997 (0.010)	5.963 (2.932)	0.009 (0.005)	**−1.508**	**−24.923**
ST22^P^	C	2016	N47°21′02.14″/E103°44′06.94″	Orkhon Province	34	19	7.816	0.954 (0.017)	6.100 (2.976)	0.009 (0.005)	−0.87	**−5.234**
ST23^P^	C	2016	N47°42′02.17″/E105°53′40.74″	Tuv Province	37	10	5.616	0.889 (0.023)	5.967 (2.911)	0.009 (0.005)	0.282	1.795
ST24^P^	E	2016	N46°06′26.3″/E108°43′20.88″	Dornogobi Province	11	11	10	1.000 (0.039)	6.436 (3.302)	0.010 (0.006)	−0.824	−5.868
China		2015	N40°56′66″/E109°92′45″	Baotou	3	2	—	0.667 (0.314)	3.333 (2.323)	0.005 (0.004)	0.000	2.357
France		1971	N42°13′53.40″/E8°37′40.66″	Corsica Island	2	2	—	1.000 (0.500)	7.000 (5.292)	0.011 (0.011)	0.000	1.946
Korea		1931–1966	N36°14′34.1″/E128°34′22.4″	Gyeongsangbuk-do	7	2	—	0.952 (0.096)	5.143 (2.834)	0.008 (0.005)	0.000	2.357
Mean							—	0.980 (0.004)	5.622 (2.703)	0.008 (0.005)	**−1.769**	**−24.643**

Significant values (*P* < 0.05) are indicated in bold.

C, Central; E, East; N, North; S1, South1; S2, South2; S3, South3;

Superscript P in locality denotes populations that were represented by more than ten samples (N > 10);

*Indicates very close localities but collection in different years.

Estimates of haplotype diversity (*h*) for the 14 large (N > 10) populations ranged from 0.889 to 1.000 in the ST23 and ST24 populations, respectively, with an average of 0.974, whereas estimates of nucleotide diversity (*π*) ranged from 0.006 to 0.010, with an average of 0.008 (Table 1). Furthermore, the number of haplotypes in these populations ranged from ten to 29 in the ST23 and ST18 populations, respectively, which were located in the central and southernmost of Mongolia. The most common haplotype (H19) was shared by 47 individuals (11.2%) and was obtained from all but seven of the localities in Mongolia (ST02, ST05, ST08, ST09, ST11, ST12, and ST18), and the next most common haplotypes, H21, H15, H12, H30, and H02, accounted for 4.5, 3.6, 2.9, 2.9, and 2.6% of the sampled individuals, respectively. Meanwhile, 181 of the haplotypes (43.4%) were obtained from single individuals and, thus, classified as unique haplotypes (i.e., singletons). Most of the localities yielded relatively high haplotype richness (HR) values, ranging from 5.616 to 10, with the lowest HR value obtained for the ST23 population, which was located in the center of our sampling localities.

### *COI* haplotype network

**Figure 2:**
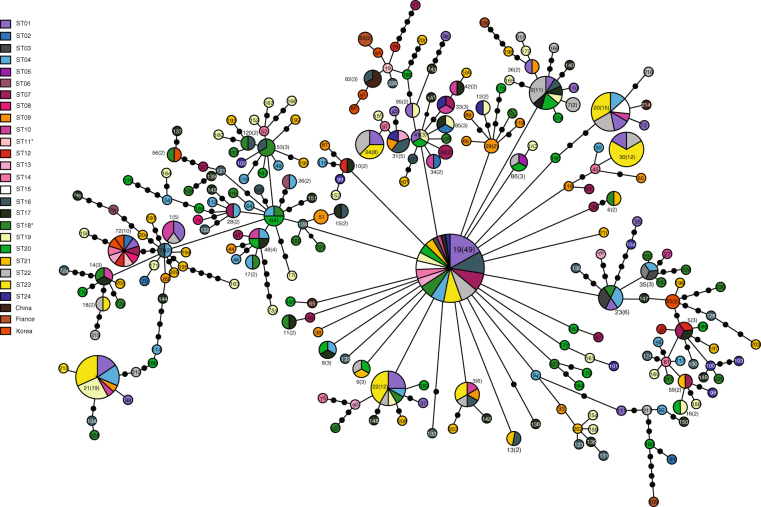
Haplotype network of *COI* sequences from 14 populations of *Gymnopleurus mopsus*. Each color represents different localities. Each line in the network denotes a single mutational step, irrespective of its length. Numbers shown either inside or outside of each circle and the accompanying numbers in parentheses denote haplotype identification numbers and the numbers of individuals belonging to each haplotype, respectively. The areas of the circles are also proportional to the number of individuals represented by each haplotype, and the small, filled circles represent intermediate haplotypes that were not present in our samples but are necessary to connect the observed haplotypes to the network.

A haplotype network of *COI* sequences was star-shaped, with the most common haplotype (H19) located in the most internal position in the network (Fig. 2), thereby indicating presumably an ancestral haplotype. Furthermore, the ancestral H19 haplotype was detected from all but seven of the sampling localities in Mongolia (ST02, ST05, ST08, ST09, ST11, ST12, and ST18), which indicates its wide distribution. Related haplotypes that radiated from H19 were found repeatedly in different parts of the network, which suggests that local population expansion was followed by radiation from the ancestral haplotype. The haplotypes of individuals from Korea (N = 7, H25, H56, H80, H81, H83, and H84) and China (N = 3, H82 and H65) were located at the edge of the network but with only one or two mutational steps separating them from the haplotypes of the Mongolia populations. Meanwhile, the two haplotypes (H105 and H106) from France (N = 2) were positioned at the tip of the network, with four or five mutational steps separating them from the Mongolia haplotypes.

### Geographic population structure

Hierarchical analyses of molecular variance (AMOVA) revealed significant spatial genetic structuring among the 24 Mongolia populations, according to three different biogeographic groupings of (1) two groups, the humid areas (North and Central populations) and desert areas (South and East populations); (2) three groups, the North and Central area (NC), South area (S), and East area (E); or 3) three groups of South populations (S1: ST06, ST07, ST08, ST09, ST10, ST11, and ST18; S2: ST12, ST13, ST19, ST20, and ST21; and S3: ST05, ST14, ST16, and ST17). These groupings were based on our *a priori* expectation of the spatial population differentiation of *G*. *mopsus*, based on geographic proximity to one another and to the region’s main roads (Table 2). We found that 3.06 and 2.17% of the molecular variance was partitioned between and among the groups, respectively (*ϕ*_*CT*_; *P* < 0.01), whereas most of the variance occurred within populations (*ϕ*_*ST*_; 96.74 and 97.60% of the total variation, *P* < 0.01; Table 2). However, only 0.20 and 0.23% of the molecular variance was partitioned among populations within groups (*ϕ*_*SC*_; *P* > 0.05). The greatest between-group difference (3.06%) was observed when the four populations (ST01, ST15, ST22, ST23) in the northern humid area were compared to the 20 desert populations. However, the genetic structure (*ϕ*_*CT*_ = − 0.002) among the three South groups (S1, S2, and S3) was insignificant (*P* = 0.775), which indicated the occurrence of relatively high levels of either historical or contemporary gene flow among populations in South area.

**Table 2:** Analysis of molecular variance (AMOVA) of *Gymnopleurus mopsus* in Mongolia.

Grouping	Source of variation	d.f.	Sum of squares	Variance components	Percentage of variation	**ϕ-**Statistics	*P* value
2 groups (humid vs. desert)	Among groups	1	16.782	0.08686	3.06	**ϕ**_**CT**_** = **0.03057	**0.007**
	Among populations within groups	22	62.473	0.00578	0.20	**ϕ**_**SC**_** = **0.00210	0.328
	Within populations	382	1049.918	2.74848	96.74	**ϕ**_**ST**_** = **0.03261	**0.003**
3 groups (NC, S, E)	Among groups	2	19.464	0.06121	2.17	**ϕ**_**CT**_** = **0.02174	**0.000**
	Among populations within groups	21	59.791	0.00644	0.23	**ϕ**_**SC**_** = **0.00234	0.316
	Within populations	382	1049.918	2.74848	97.60	**ϕ**_**ST**_** = **0.02402	**0.003**

Significant *P-*values (*P* < 0.05) are indicated in bold.

### Genetic differentiation (*Fst*) and isolation by distance (IBD)

**Figure 3:**
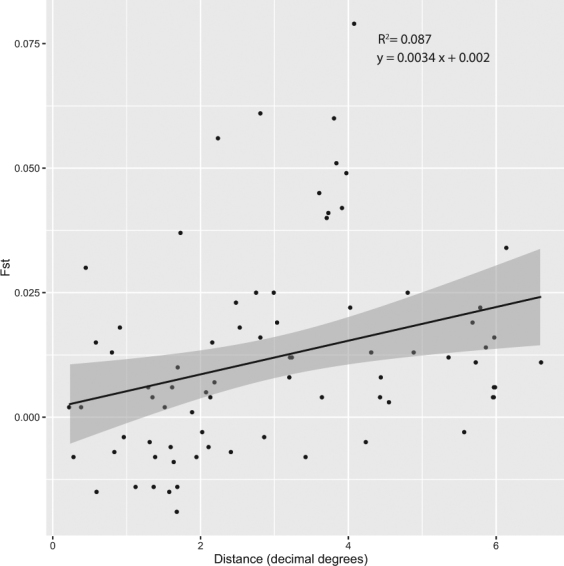
Relationship between the genetic distance (*Fst*) and geographic distance (decimal degree) of paired populations. These two distance measures are significantly correlated (Mantel *R*^2^ = 0.0869 *P* = 0.001).

The genetic differentiation between populations was estimated using pairwise *Fst* statistics (*ϕ*_*ST*_), which ranged from −0.019 to 0.079 (Table 3). Most of the pairwise differences (*ϕ*_*ST*_ values) were relatively small, even between distant populations (e.g., the southernmost and northernmost sampling localities, which were separated by 670 km), which generally indicated low levels of inter-population genetic differentiation. As a result, after sequential Bonferroni correction applied, only 18 of the 91 comparisons among the 14 large (N > 10) populations indicated significant differentiation (*P* < 0.05; Table 3). The highest *ϕ*_*ST*_ value was observed between the ST23 and ST18 populations (*Fst* = 0.079, *P* < 0.01). However, neither the ST07 nor ST10 population was significantly differentiated from any of the other populations, which suggests the occurrence of either historical or ongoing gene flow between these and the other populations. Isolation-by-distance (IBD) analysis detected a significant positive relationship between genetic distance (*Fst*) and geographic distance (decimal degrees; R^2^ = 0.087, *P* = 0.001) between the 14 large Mongolia populations (Fig. 3).

**Table 3:** Pairwise *Fst* values of *Gymnopleurus mopsus* populations in Mongolia.

*Fst*	ST01	ST04	ST07	ST09	ST10	ST16	ST17	ST18	ST19	ST20	ST21	ST22	ST23	ST24
ST01	0.000													
ST04	0.006	0.000												
ST07	0.006	0.003	0.000											
ST09	0.011	0.004	0.018	0.000										
ST10	−0.003	0.008	0.004	0.030	0.000									
ST16	0.013	−0.005	−0.019	0.006	0.010	0.000								
ST17	0.013	0.004	−0.014	0.006	0.002	−0.015	0.000							
ST18	**0.034**	0.012	−0.007	**0.037**	0.015	−0.008	−0.014	0.000						
ST19	**0.014**	**0.019**	−0.005	0.004	0.018	0.001	−0.008	0.015	0.000					
ST20	0.004	0.004	−0.015	−0.007	0.016	−0.006	−0.009	0.013	−0.008	0.000				
ST21	**0.022**	0.016	−0.014	**0.023**	−0.004	−0.003	−0.006	−0.004	0.002	0.002	0.000			
ST22	−0.008	0.011	0.022	0.013	0.008	**0.025**	0.019	**0.041**	0.012	0.012	0.025	0.000		
ST23	0.005	**0.025**	0.042	0.040	0.045	**0.056**	**0.061**	**0.079**	**0.051**	**0.049**	0.060	0.007	0.000	
ST24	0.045	**0.047**	−0.007	**0.053**	0.050	0.022	0.010	0.026	−0.001	0.033	**0.030**	**0.043**	0.075	0.000

Significant Bonferroni-corrected *Fst* values (*P* < 0.05) are indicated in bold.

### Demographic history

**Figure 4:**
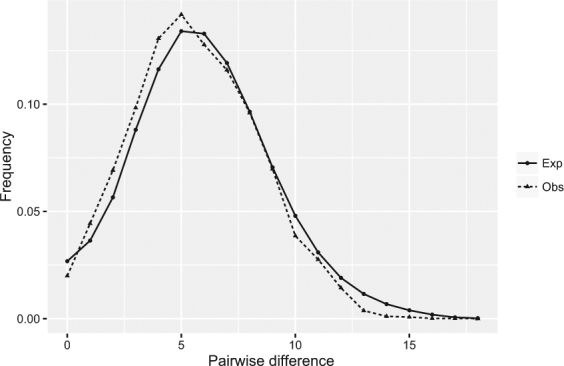
Mismatch distribution of nucleotide differences between pairs of mtDNA haplotypes. There were no significant differences between the observed and expected frequencies (sum of squared deviation = 0.000344, *P* = 0.75).

Phylogeographic analyses revealed a recent, sudden expansion of *G*. *mopsus* populations, based on several lines of evidence, including neutrality tests and the mismatch distribution analysis (Table 1, Fig. 4). For example, both the Tajima’s *D* and Fu’s *Fs* statistics for neutrality were negative for all but the ST23 population, which was located in the central area of all the main roads meet in Mongolia (Fig. 1). Moreover, the neutrality test was significantly violated when the all of the 14 populations were pooled as a single data set (Table 1), with Tajima’s *D* and Fu’s *Fs* values of −1.76936 (*P* = 0.005) and −24.64263 (*P* = 0.001), respectively, which suggests that a sudden historical population expansion occurred following a population bottleneck. These significant negative values also indicate that the hypotheses of population stasis, selective neutrality, and population equilibrium can be rejected.

A mismatch distribution was clearly smooth and unimodal for all of the 14 large populations, further supporting the model of sudden population expansion^18^ (Table 1, Fig. 4). The SSD (sum of squared deviations) statistic indicated that the observed frequency distribution fit the distribution expected under the model of demographic expansion (SSD = 0.00037, *P* = 0.92, raggedness index 0.00604, *P* = 0.77), and both the non-significant SSD statistic and small raggedness index indicated sudden population expansion, again confirming the results of the neutrality tests, whereas the non-significant raggedness index indicated a good fit to a model of population expansion^19^ (under the demographic expansion model). Based on the pooled Mongolia populations, the mismatch distribution again fit the model of demographic expansion.

**Figure 5:**
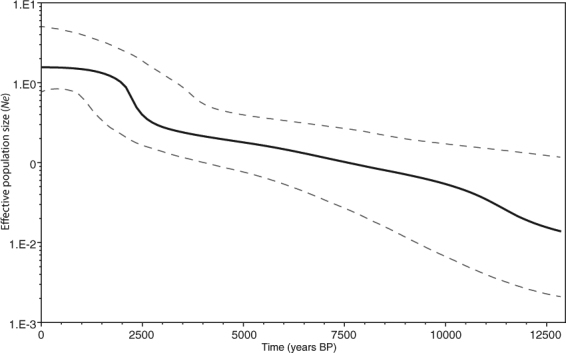
Bayesian skyline plot of *COI* sequences from *Gymnopleurus mopsus*. The black solid line indicates the median effective population size, and the dashed line indicates the range of 95% highest posterior density (HPD) intervals.

Historical demographic reconstructions, based on Bayesian skyline plot (BSP) analysis, further supported a model of sudden population expansion (Fig. 5). Indeed, the Mongolia populations appear to have undergone a population expansion ~2500 years ago, followed by continuous increases in effective population size for a long period afterward (Fig. 5). This estimate might indicate that the timing of *G*. *mopsus* expansion corresponded to both the expansion of human populations and advent of large mammal domestication in Mongolia.

## Discussion

To the best of our knowledge, the present study is the first to examine the current population genetic structure and historical population demography of *G*. *mopsus* spanning major distributional ranges across Mongolia using mitochondrial *COI* sequences. The high frequency of unique haplotypes (singletons) and limited nucleotide differences between haplotypes could reflect rapid and recent population expansion, following a population bottleneck. Otherwise, such rare mtDNA variants would be eliminated by genetic drift. The significant negative Tajima’s *D* and Fu’s *Fs* statistics of the pooled *G*. *mopsus* populations also reject the hypothesis of the constant population size assumed under mutation-drift equilibrium^20^. In addition, the star-shaped topology of the haplotype network is another line of evidence that suggests the sudden demographic expansion of dung beetles, as in previous studies on flies^21,22^, moths^23^, leaf beetles^24^, and marine gastropods^25^, as well as domestic cattle and goats^26,27^. The most common and presumably ancestral *G*. *mopsus* haplotype (H19) was located in the center of the haplotype network and was connected to a large number of infrequent haplotypes by only a few mutational steps. This pattern is generally interpreted to indicate a population that has recently expanded in size from a small number of founders following a population bottleneck^28^. The single bell-shaped mismatch distribution for the *COI* haplotypes also supported the hypothesis of population range expansion^29^, whereas the hypothesis of sudden population expansion was further supported by the abrupt increase in effective population sizes, as suggested by BSP analysis (Fig. 5). Thus, all the analyses of *COI* sequences from *G*. *mopsus* in Mongolia indicated sudden population expansion.

The historical demographic expansion of domesticated animals, which has resulted from archeological or anthropogenic events (e.g., domestication), has been well documented in a number of taxa^21,27,30,31^. We speculate that the recent population expansion observed in *G*. *mopsus* is related to the population dynamics (e.g., expansion) of large mammals, such as cattle, horses, and camels, that provide food and breeding ground for *G*. *mopsus* and, therefore, limit its abundance and distribution. The expansion of *G*. *mopsus* populations is estimated to have occurred ~2500 years ago, based on BSP analysis with estimated substitution rate. Such estimates depend on which molecular clock rates are applied, since different lineages are often subject to different mitochondrial substitution rates^32^. However, the hypothesis of a recent history of expansion was supported by many other analyses, as well, and the population expansion of *G*. *mopsus* was estimated to have occurred a few thousand years ago, based on the BSP analysis that accounts for the variation in our time estimation. Interestingly, our estimated timing of *G*. *mopsus* expansion seems to correspond to the advent of livestock domestication in Mongolia. Thus, the domestication of cattle and livestock was a turning point in the histories of both human and dung beetle populations^33^. The earliest domestication of wild cattle in northeastern Asia, including Mongolia, North China, Korea, and Japan, has been suggested to have occurred between 4000 and 5000 years ago^34^. The similar population structure of livestock and *G*. *mopsus* in Mongolia might further support the hypothesis of livestock-mediated expansion of *G*. *mopsus* populations. Recent population expansion in various domesticated animals, including cattle^26^ and goats^27,35^, has been supported by bell-shaped mismatch distributions or star-shaped haplotype networks, much like the ones presented for *G*. *mopsus* populations in the present study. Furthermore, other population genetics studies of cattle populations have also reported unusually high genetic diversity within domesticated populations in Mongolia^35^, as well as domestication-driven population expansion^34^. Moreover, such studies have also observed that the most common and presumably most ancestral haplotypes are located at the centers of the haplotype networks and that a number of rare haplotypes originate from that haplotype with only a few mutational steps.

In a previous study of cattle dung-using *Helictopleurus* species (*H*. *neoamplicollis* and *H*. *marsyas*) in Madagascar, researchers used *COI* sequences to document a rapid population expansion that followed a resource shift^36^. The resource shift from primate droppings to cattle dung resulted in extended geographic ranges and population expansion with low genetic diversity and was associated with the historic introduction of cattle ~1500 years ago^36^. Indeed, the two *Helictopleurus* species exhibited considerably lower *COI* haplotype diversity than more wild, forest-dwelling dung beetles with more varied diets^36^. Another common cattle dung-using species, *H*. *quadripunctatus*, possesses a more intermediate diet, in that it is less specific, and, compared with forest-dwelling species, exhibits more similarities to the *G*. *mopsus* populations in Mongolia, particularly in regards to its high haplotype diversity, low nucleotide diversity, and significant IBD. In fact, like *H*. *quadripunctatus*, *G*. *mopsus* feeds on a broad range of food sources (e.g., sheep and camel dungs). Thus, a resource shift by *G*. *mopsus* from wild animal dung to domesticated cattle droppings during the domestication of cattle in Mongolia could account for the species’ demographic expansion. The contemporary cattle population in Mongolia is known to have originated from hybridization with wild yak populations ~7300 years ago and has probably undergone rapid population expansion for the last 3600 years^37^.

The dispersal potential (dispersal capacity) of a species is a critical factor in characterizing its present-day population genetic structure. There is evidence that the dispersal and movement patterns of dung beetles are strongly associated with those of mammals^13,14^. Overall, the weak genetic structuring observed among the *G*. *mopsus* populations of Mongolia, as indicated by low *Fst* values, suggests the occurrence of either contemporary or historical gene flow. In particular, the significant relationship between the genetic and geographic distances of population pairs (IBD) indicates that the geographic proximity of populations significantly influences the spatial genetic structure of *G*. *mopsus*, probably resulting from the fact that its dispersal ability is associated with the geographic closeness.

The movement processes of dung beetles might be stochastic since individuals could move short distances between dung pats, as well as long distances between pastures, during foraging. Maximum flight distance of 1.5–2 kilometers per day has been reported in a previous study^38^. Thus, the dispersal and migration distances of this small beetle are likely limited, especially when considering the long distances between certain populations (e.g., ~670 km). However, if the migration of *G*. *mopsus* is promoted by other factors, such as livestock dispersal (i.e., herd movement during pasturing), the high level of connectivity between the *G*. *mopsus* populations becomes much more conceivable. Pasturing is a popular type of livestock farming (i.e., nomadic livestock husbandry) in Mongolia, and farmers often move their cattle herds among grasslands on a seasonal basis, following road networks. Such long-distance dispersal, either by long-distance flight or long-distance animal-mediated movement, could explain the low levels of inter-population genetic differentiation observed in the present study.

The AMOVA indicated weak, but detectable genetic structuring among certain *G*. *mopsus* population groups (i.e., North and Central, East, South area) that were clustered by the major roads that are seasonally used for moving cattle (Table 2). However, the species’ complicated dispersal system (i.e., short-distance foraging movement and long-distance dispersal), which is the result of dung availability and possibly other biological factors, could lead to variability in the species’ dispersal. Therefore, further investigation *of G*. *mopsus* foraging behavior and dispersal potential is needed, especially elucidate the influence of dung beetle dispersal on population connectivity in Mongolia.

In the present study, we also identified an extraordinarily high level of genetic diversity, which could indicate extremely large effective population sizes (*N*_e_) and, possibly, high levels of historical and ongoing gene flow. Large *N*_e_ values typically indicate that populations have undergone historical demographic expansion but are now stable, thereby suggesting that *G*. *mopsus* originated in Mongolia. Our haplotype network analysis, which included individuals from multiple regions, including South Korea, China, and France, also supports the hypothesis that *G*. *mopsus* originated in Mongolia. Indeed, the haplotypes from South Korea and China were connected to those from Mongolia by only one or two mutational steps, and the haplotypes from France were not much more removed. It is possible that Mongolia is the geographic origin of *G*. *mopsus* expansion and that the species radiated (or migrated) to both the East and West during the demographic processes of population expansion.

The high genetic diversity of the remnant *G*. *mopsus* populations in Mongolia might allow the species to adapt to changes in local environments, including changes in climate, vegetation, and dung availability. Mongolia includes both arid and semi-arid regions, where climate change is generally more drastic than in other regions^17^. In particular, the largest genetic differentiation was detected between populations from the northern humid areas (grassland or forest-steppe areas) and southern dessert area, which were characterized by different vegetation types, i.e., grassland or forest-steppe areas and desert, respectively. A previous study^39^ reported that there was substantial year-to-year variation in the insect community of Mongolia. For example, in one year, the number of coleopteran species at a region near our ST23 locality was reduced by 60%^39^. The ST23 population, which was located at the center of a main road and was the most urbanized of our sampling localities, possessed the lowest genetic diversity of the Mongolia populations and yielded only negative Tajima’s *D* and Fu’s *Fs* values. This could indicate urbanization effects, which would indicate that the species is at a high risk of population size reduction in Mongolia. However, the widespread *COI* haplotypes and the pattern of star-shaped haplotype network may still indicate that the species is highly adaptive. Therefore, in order to understand the relationship between the genetic diversity and ecological adaptive potential of *G*. *mopsus*, future studies should investigate the response of *G*. *mopsus* to diverse environmental dynamics.

To design effective conservation and restoration management plans for *G*. *mopsus* in South Korea, understanding the genetic characteristics (e.g., genetic diversity, genetic structure, and phylogenetic or phylogeographic relationships) of potential source populations, like those of Mongolia, will be crucial^40^. The National Institute of Biological Resources in South Korea established a restoration program in 2012. The conservation and restoration efforts of endangered and regionally extinct species often require the introduction of individuals from source populations, whose genetic and evolutionary lineages are comparable to those of the recipient populations. Therefore, both the population genetic structure and phylogenetic relationships of source populations should be considered carefully when developing reintroduction programs^41^. The findings of the present study highlight the value of the Mongolia populations for *G*. *mopsus* conservation and suggest that the populations could be used as source populations for restoration efforts in South Korea. Additionally, this species has been suggested to have a very large distributional range in Palearctic region^42^. Therefore, the observed phylogeographic trends by molecular data in this study need to be corroborated in a wider spectrum of its geographic distribution in future research, and it will also allow for exploring the conservation values of other remnant and potentially important populations of this species.

The genetic data presented by the present study provide valuable information about the phylogeographic structure, evolutionary history, and the genetic structure of *G*. *mopsus* populations across Mongolia. Additional ecological studies could further contribute to the effectiveness of dung beetle restoration in South Korea. The results of the present establish a basis for future research regarding the effective conservation and restoration of this highly cherished dung beetle species.

## Methods

### Sample collection, DNA extraction, and mtDNA *COI* sequencing

Between 2014 and 2016, a total of 406 adult *G*. *mopsus* individuals were collected from 24 localities in Mongolia. Upon collection, the specimens were washed with distilled water to remove dirt and animal dung and then stored in absolute ethanol until subject to DNA extraction. Dried specimens that were collected from South Korea from 1931 to 1964 (N = 7), France in 1971 (N = 2) and China in 2015 (N = 3) were also included (Table 1) because the populations in those regions are now regionally extinct. Especially, the species in the western part of Europe (e.g., France) is almost extinct or in great danger of extinction compared to one in the eastern part of Europe (e.g., Greece)^43^.

Genomic DNA was isolated from a femur of each specimen using the DNeasy Blood & Tissue Kit (Qiagen, USA), and polymerase chain reaction (PCR) was used to amplify a 658 bp fragment of the mitochondrial cytochrome oxidase I gene (*COI*), using 20-μl reactions, universal forward (LCO1490: 5-GGT CAA CAA ATC ATA AAG ATA TTG G-3)^44^ and reverse (C1-N-2191: 5-CCC GGT AAA ATT AAA ATA TAA ACT TC-3)^45^ primers using AccuPower PCR Premix (Bioneer, Korea), with the following conditions: an initial denaturation at 94 °C for 1 min; followed by 35 cycles of 30 s at 94 °C, 30 s at 50 °C, and 1–2 min at 72 °C; and a final extension step of 72 °C for 7 min. The PCR products were visualized on 1.5% agarose gels using UV light, purified enzymatically using Exonuclease I and Shrimp Alkaline Phosphatase (New England BioLabs, USA), and then sequenced by Macrogen INC Sequencing (Korea) using an ABI PRISM 3130xl Genetic Analyzer (Applied Biosystems, USA). The resulting sequences were deposited in GenBank under accession numbers MF674025–MF674381. Previously published *COI* sequences collected in South Korea from 1931 to 1964 (KT454107-454111, KX879011-879012) and from Mongolia (KT454096-454106, KX879013-879046, KX879050-879055) and China (KX879047-879049) were also included^12^.

### Haplotype network and genetic diversity analyses

The *COI* sequences were aligned using Clustal W in BioEdit v.7.0.1^46^ and checked manually, and the haplotypes of the 418 individuals were determined using Neighbor-Joining algorithms in MEGA 7.0^47^. A haplotype network was then inferred using HAPSTAR v0.7^48^.

The total and population genetic diversity indices, including the number of haplotypes (HN), haplotype diversity (*h*), and nucleotide diversity (π), were estimated using ARLEQUIN v3.5^49^, and Tajima’s *D* and Fu’s *Fs* statistics were also analyzed to test for neutrality. Ten populations that had insufficient sample sizes (N < 10) were excluded from all subsequent analyses, except the AMOVA. Haplotype richness was calculated using a rarefaction method in CONTRIB v1.02^50^ that corrects for unequal sample sizes among populations.

### Population genetic differentiation and isolation by distance (IBD)

The fixation index (*Fst*)^51^ was calculated for population pairs using ARLEQUIN v3.5, and the geographical distance between sampling localities was measured in decimal degrees using ArcGIS Desktop 10.5 (ESRI, USA)^52^. The correlation between genetic distance (*Fst*) and geographic distance (decimal degrees), i.e., the Mantel test (spatial autocorrelation multivariate approach)^53^, was used to test for isolation by distance (IBD) in GenAlEx v6.502^54^.

### Geographic population structure

A hierarchical analysis of molecular variance (AMOVA) was performed using ARLEQUIN v3.5, in order to assess the spatial population genetic structure of *G*. *mopsus* across its main range in Mongolia. Each of the 24 populations (Table 1) was assigned to one of four biogeographic areas, i.e., North (N), Central (C), South (S), or East (E), following the major road networks in Mongolia. For this analysis, the populations were either separated into either (1) two groups, the northern humid areas (North and Central populations) and desert areas (South and East populations); (2) three groups (NC, S, and E), which were classified based on the major roads (N: ST01, ST15, ST22, and ST23 populations; S: ST05, ST06, ST07, ST08, ST09, ST10, ST11, ST12, ST13, ST14, ST16, ST17, ST18, ST19, ST20, and ST21 populations; and E: ST02, ST03, ST04, and ST24 populations); or (3) three groups (South populations only; S1: ST06, ST07, ST08, ST09, ST10, ST11, and ST18 populations; S2: ST12, ST13, ST19, ST20, and ST21 populations; and S3: ST05, ST14, ST16, and ST17 populations; Fig. 1, Table 2). Total molecular variance was partitioned among groups (Fct = ‘inter-group’ genetic variation), populations within groups (Fsc = ‘intra-group’ genetic variation), and populations, regardless of groupings (Fst = ‘inter-population’).

### Historical demography

The expansion of *G*. *mopsus* populations was tested using mismatch distribution analysis and both Tajima’s *D*^55^ and Fu’s *Fs* neutrality tests^56^ in ARLEQUIN v3.5. Meanwhile, the demographic history of *G*. *mopsus* in Mongolia was determined using Bayesian Skyline Plot (BSP) analysis in BEAST v2.4.5^57^. The GTR + G + I model of evolution was selected as the best model by jModeltest^58^. MCMC run was performed with 300 M iterations, with sampling every 1000 iterations, the first 10% of generations discarded as “burn-in”, a constant skyline model, and Bayesian skyline tree priors. The molecular clock rate and substitution rates were estimated as a default function. The run was examined using Tracer v.1.6^59^, and all the parameters were analyzed to confirm that the effective sample sizes were sufficient (ESS > 200).
